# Utilization of Reconstituted Whey Powder and Microbial Transglutaminase in Ayran (Drinking Yogurt) Production

**DOI:** 10.17113/ftb.60.02.22.7383

**Published:** 2022-06

**Authors:** Ceren Akal, Celalettin Koçak, Nazlı Kanca, Barbaros Özer

**Affiliations:** Department of Dairy Technology, Faculty of Agriculture, Ankara University, Sehit Omer Halisdemir Cad., 06110, Diskapi, Ankara, Turkey

**Keywords:** fermented products, whey, transglutaminase, SDS-PAGE, volatile profile, rheology

## Abstract

**Research background:**

In industrial ayran production, milk is diluted to the desired protein content (2% (*m*/*V*)) prior to fermentation by yogurt starter cultures by partial replacement of cheese whey in reconstituted form with potable water. This may be an alternative way of protein recovery from cheese by-products as well as reducing the production costs since less milk is used in ayran production. On the other hand, the balance between milk caseins and whey proteins is disturbed when cheese whey is added to milk for ayran production, which likely leads to a time-dependent phase separation during cold storage. Modification of ayran matrix by enzymatic crosslinking of proteins may be a solution to overcome this potential physical instability of ayran. This topic has not been explored to date, and the present study was designed to investigate the possibilities of utilization of reconstituted whey powder (RWP) and microbial transglutaminase (MTG) in ayran production.

**Experimental approach:**

Milk was diluted to obtain 2% protein level using RWP and potable water. The aim of utilization of RWP was to meet 5, 10 or 15% of the protein content of the final product. RWP solutions were obtained by calculating the amounts of whey powder required to meet the specified ratios and mixing it with the water required for dilution. We prepared eight different ayran samples divided in three groups, namely group A: prepared by partially diluting milk with RWP to obtain 5, 10 or 15% of total protein amount in the product, group A_MTG_: prepared by adding microbial transglutaminase (0.5 U per g of protein) to group A samples, and control group without RWP and with or without the addition of MTG. The gross composition, physical (phase separation and viscosity, chemical (volatile and peptide profiles and SDS-PAGE electrophoresis patterns) and sensory properties of the samples were evaluated throughout 15 days of storage with weekly intervals.

**Results and conclusions:**

Since the amounts of whey powder used to obtain RWP were different, dry matter levels of the samples differed. Using RWP in ayran production increased the phase separation slightly. Incorporation of MTG affected the physical properties of the ayran samples positively and prevented phase separation at a satisfactory level. SDS-PAGE electrophoretograms revealed that cross-linking between proteins triggered by MTG formed intense bonds at high molecular mass regions. The remaining parameters were not affected by MTG. Results revealed that the samples with 10% RWP with and without addition of MTG were determined as the formulations with the highest commercialization potential.

**Novelty and scientific contribution:**

Utilizing RWP in the production of ayran to reduce the protein content of the final product to the desired level is a new approach. Since complete replacement of RWP with potable water to dilute milk to reach 2% (*m/V*) protein likely leads to lower sensory scores, we have investigated a possibility of partial replacement of RWP with potable water. A time-dependent phase separation is the major challenge of industrial ayran production. This physical problem was largely eliminated by means of MTG-mediated cross-linking of milk proteins. The proposed novel ayran production method offers dairy industry reduction of production costs and contributes to sustainability in milk production since smaller volume of milk is used to reach desired protein content in the final product.

## INTRODUCTION

Ayran is a fermented milk beverage which is popular in Turkey with annual production volume of 730 582 tonnes (*1*). This fermented beverage is also widely consumed in some Balkan countries, Central Asia, Iran (under the name of doogh) and the Middle East (*2*). Ayran is produced either by dilution of yogurt with potable water (~1:1 dilution in traditional practices) or by fermentation of pre-diluted milk by yogurt starter bacteria (in industrial practices). According to Turkish Food Codex, the product must have minimum of 2% (*m*/*V*) milk proteins (*3*). The typical shelf life of ayran is about two to three weeks at 4 °C. If high homogenization technique is used in ayran production (35 000-40 000 kPa), the physical stability of the product can be ensured for a longer time (up to four weeks. Additionally, the choice of starter culture is another factor affecting the shelf life of ayran. In general, yogurt starter combinations with low post-acidification capacity are preferred in ayran production (*2*).

Cheese whey is a by-product of cheese-making and contains most of the water-soluble compounds of milk. It is estimated that annually ~11.5 million tonnes of cheese whey is produced in Turkey (*4*). Vast majority of this amount is converted to whey powder, whey protein isolate/concentrate and a small quantity of cheese whey is utilized to produce lactose in liquid and powder forms. Large-scale dairy companies convert cheese whey into value-added products at their facilities, but medium- and small-sized dairy companies do not have such a capacity. Therefore, the development of novel strategies or approaches to utilize cheese whey is of importance especially for small- and/or medium-scale dairies (*5*).

The use of sweet cheese whey in the industrial ayran production offers an option for cheese whey valorisation. In addition, incorporation of cheese whey into the manufacture of ayran as partial substitution of milk has a potential of reducing production costs.

The major challenge of ayran production is the time-dependent separation of liquid and solid phases as the use of stabilizing agents in ayran production is prohibited in Turkey. In order to overcome this problem, a number of manufacturing strategies have been developed. The incorporation of microbial transglutaminase (MTG, γ-glutamyltransferase, EC 2.3.2.13) into the milk for ayran production was demonstrated to be a successful way of improving physical quality of the product (*6*, *7*). The MTG successfully improves the physical stability of dairy products by catalysing the formation of crosslinking between the amine groups of proteins (*i.e.* glycine) and the γ-carboxyamide residues of glutamine with acyl transfer. Additional covalent bonding formed by MTG-triggered crosslinking results in higher viscosity and lower whey separation in dairy products (*6*). In addition to the economic benefits provided by partial replacement of milk with whey in ayran production, to the best of our knowledge, there is no research conducted to overcome possible physical defects (*i.e*. phase separation) in the final product.

There is a limited number of studies on the use of whey and/or transglutaminase in ayran production (*7,8*). Rezazadeh-Bari *et al*. (*8*) showed that in probiotic ayran production, milk replacement with whey protein up to 70% along with MTG treatment (5 U/100 mL) resulted in an acceptable end product regarding physical stability and viability of probiotic strains. The same authors demonstrated that the replacement of more than 70% milk with whey protein triggered sedimentation. Improvement of physical stability of ayran under cold storage in the presence of MTG (1 U per g milk protein) was also reported by Şanli *et al*. (*7*).

The present study aims to investigate the possibilities of partial replacement of potable water by reconstituted whey powder in the manufacture of ayran. The use of water only instead of reconstituted whey powder in ayran production is not possible due to sensory defects. Instead, the possibility of adding reconstituted cheese whey powder at a level to ensure that the targeted protein content of ayran is met at certain values has been investigated. To achieve this goal, milk used for ayran production was diluted with the solution of reconstituted whey powder (RWP) to obtain 5, 10 or 15% of total protein content of the resulting product and then converted to ayran in the presence or absence of MTG. This approach aims both to create a new area of ​​use of cheese whey and to contribute to sustainability in milk production by using a smaller volume of milk in the manufacture of unit volume of ayran.

## MATERIALS AND METHODS

### Materials

Raw bovine milk with the following gross composition (%, *m*/*V*): dry matter (12.1±0.2), fat (3.60±0.07), protein (3.5±0.2), ash (0.61±0.03) and lactic acid (0.16±0.01), at pH=6.74±0.04 was supplied from the Dairy Pilot Plant of Ankara University (Ankara, Turkey). Cheese whey powder with the following gross composition (*w*/%): moisture (0.89±0.01), fat (0.65±0.03), protein (6.65±0.07) and ash (5.12±0.01) was obtained from Enka Milk and Food Products Inc. (Konya, Turkey). Fermentation was achieved by using yogurt starter culture containing *Streptococcus thermophilus* and *Lactobacillus delbrueckii* ssp. *bulgaricus* (YO-FLEX YC X16; Chr. Hansen Co. Inc., Hørsholm, Denmark). The culture is characterized with texture-enhancing capacity and fast acidification properties, as declared by the manufacturer. Microbial transglutaminase (Activa® MP) was kindly supplied from Ajinomoto Istanbul Gida Ltd. (Istanbul, Turkey). All the chemicals were of analytical grade and supplied from Sigma-Aldrich, Merck (St. Louis, MO, USA) unless otherwise stated.

### Ayran production

**Fig. S1:**
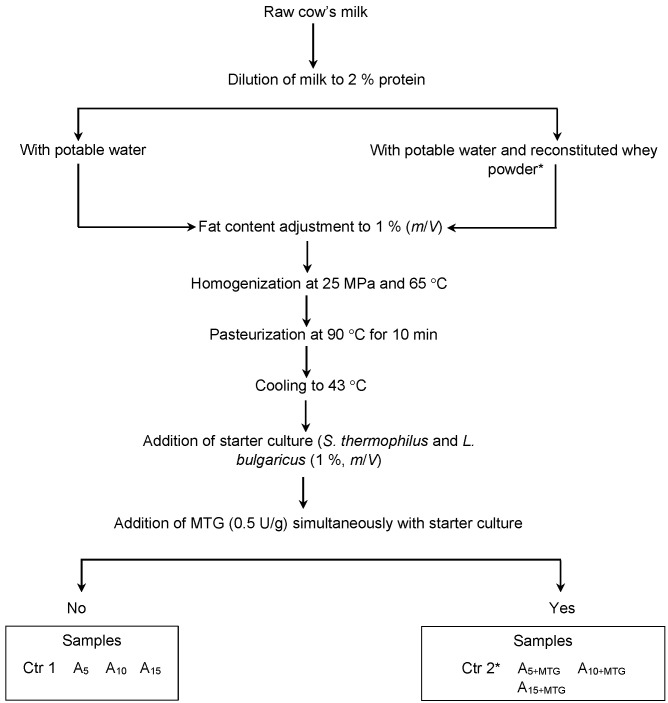
Schematic illustration of experimental ayran production. Ctr 1 and 2=samples without RWP and MTG (1) and with MTG (2) as control; A_5_, A_10_ and A_15_=samples with 5, 10 and 15% RWP; A_5+MTG_, A_10+MTG_ and A_15+MTG_=samples with 5, 10 and 15% RWP and 0.5 U/g MTG, RWP=reconstituted whey powder, MTG=microbial transglutaminase

Ayran samples were produced by fermenting diluted milk according to Özer (*2*). Milk was diluted with combinations of reconstituted whey powder (RWP) and potable water. According to Turkish Food Codex, ayran must have a minimum of 2% (*m*/*V*) protein (*3*). Therefore, milk must be diluted to reduce protein level. In the present study, dilution of milk was achieved either by adding potable water (control sample) or by adding RWP. The volume of added RWP solution was calculated to achieve the substitution of 5, 10 or 15% of milk proteins, indicating that 0.1, 0.2 or 0.3% protein in the final product came from RWP, respectively. To do this, the amount of whey powder to meet 5, 10 or 15% of the final protein level (2%) was calculated and this amount was reconstituted and added to milk. The diluted raw milk was standardized to 1% (*m*/*V*) fat and then homogenized at 25 MPa and 65 °C. The pre-diluted milk was heated at 90 °C for 10 min and then cooled to 43 °C. Afterwards, heat-treated milk was divided into eight samples. The first three were inoculated with only 1% (*m*/*V*) yogurt starter culture (samples A_5_, A_10_ and A_15_) and the second three were inoculated simultaneously with yogurt starter culture and microbial transglutaminase (MTG) (samples A_5+MTG_, A_10+MTG_ and A_15+MTG_). The amount of added MTG was determined after preliminary trials. Based on the sensory evaluations and physical stability against phase separation, 0.5 U MTG per g protein gave the most acceptable results (Table S1). Hence, for experimental studies we used 0.5 U MTG per g protein. The seventh sample (positive control did not contain MTG or RWP, and the eighth sample (negative control) contained only MTG but not RWP. Incubation was achieved at 43 °C until pH=4.5 was reached. Ayran samples were then cooled to 20 °C and 0.5% (*m*/*V*) table salt was added. The samples were stored for fifteen days at 4 °C. Flow diagram of ayran production is given in Fig. S1 and the sample descriptions are given in Table S2.

### Chemical analyses

The dry matter and ash contents of samples were analysed by gravimetric method (*9*). Gerber method (*10*) was used to measure fat values using centrifugation (Nova Safety; Funke Gerber, Berlin, Germany). Total protein contents were determined by the Kjeldahl method (*11*) using digestion (model K435; Büchi, Zürich, Switzerland) and distillation unit (model 323; Büchi). The salt content was determined by titration (*12*). Titratable acidity values were also determined and expressed as the lactic acid (in %) (*13*). The pH was measured with a combined electrode digital pH-meter (model HI 83141; Hanna Instruments, Woonsocket, RI, USA). Phase separation was determined by measuring the spontaneous syneresis *as per* Lucey *et al.* (*14*) with some modifications. A volume of 250 mL of ayran samples was transferred into a volumetric flask and the volume of separated whey was measured for 15 days at weekly intervals at 4 °C.

### Microbiological analyses

Serial dilutions of ayran samples were prepared by using Ringer’s solution. *S. thermophilus* and *L. bulgaricus* colonies were counted on M17 agar (Merck Co., Kenilwoth, NJ, USA) and De Man, Rogosa and Sharpe (MRS) agar (Merck Co.), respectively. The M17 agar plates were incubated aerobically at 37 °C for 72 h and the MRS agar plates were incubated under microaerophilic conditions at 43 °C for 72 h (*15*). Microaerophilic conditions were achieved by means of an anaerobic kit (Anaerocult A®; Merck-Millipore, Burlington, MA, USA).

### Colour analyses

The colour of the ayran samples was examined by Precise Colour Reader TCR 200 (Time Group Inc., Beijing, PR China). Colour data of the samples were determined in the CIE (Commission Internationale de l'Eclairage) L*a*b* optical system (L*=lightness, a*=red-green, and b*=yellow-blue). Furthermore ∆*E* and *C* (chroma) values of samples were calculated as follows:



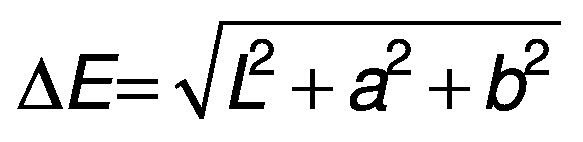





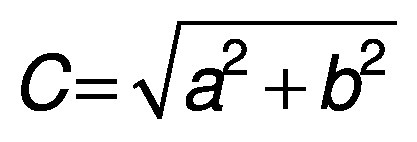



### Rheological analyses

Steady shear viscometry was done using a rheometer (Kinexus Pro+; Malvern, Worcestershire, UK) with a stainless steel 4° conical geometry spindle. Prior to rheological measurements, ayran samples were stirred using a magnetic stirrer for 30 s at constant speed. After loading, the samples were equilibrated for 30 s. Shear stress was measured at 36 logarithmically spaced shear rates in the range of 0.1-300 s^-1^ at 5 °C. The flow curves from viscometry were fitted to the power law model using non-linear regression:

*σ* = *K*⋅γ*n* /3/

where *σ* is shear stress (Pa), *K* is consistency index (Pa⋅s^n^), *γ* is shear rate (s^-1^) and *n* is flow behaviour index (dimensionless).

### Determination of volatile compounds

Volatile compounds of the samples were determined by solid phase microextraction (SPME) method. A sample mass of 5 g was weighed in vials and 10 μL of internal standard (8.1 mg/L; 2 methyl-3 heptanone and 2-methyl pentanoic acid) were added into each vial. The vials were held at 50 °C for 30 min to reach equilibrium of volatile compounds in the headspace by continuous stirring. The volatile compounds were extracted by injecting 75 μm of carboxen/polydimethylsiloxane SPME fibre (Supelco®, Sigma-Aldrich Co., Merck, Bellefonte, PA, USA) into the vial (*16*). Then, the volatile compounds were desorbed by direct insertion of the fibre into the injection port of the gas chromatograph (GC) coupled to a mass spectrometer (7890 GC-5975 MSD system; Agilent Technologies, Santa Clara, CA, USA). The GC was equipped with a DB-Wax column (30 m, 0.25 mm×0.25 μm; Agilent Technologies). Helium was used as a carrier gas at a flow rate of 1 mL/min and scan mode was 35-500 *m*/*z* at 3.12 scan/s. The initial temperature program was run at 40 °C for 10 min, followed by the increase of the temperature to 110 °C at a rate of 5 °C/min and to 250 °C at a rate of 10 °C/min.

Volatile compounds were identified by comparison of their retention times and mass spectra with those of the Wiley Registry (*17*). The amounts of volatile compounds (*N*(minimum quality)=50, which means that there are at least 50 library spectra with the largest number of peaks in common with the unknown spectrum, consistent with the minimum number of defined spectra required) were determined by the calculation of the peak area of the internal standard to the area of the unknown compound. The limits of detection (LOD; three times the relative standard deviation of the analytical blank values) were determined as >1.5 for all volatile compounds.

### Electrophoresis analysis

The changes in casein and serum protein fractions of ayran samples as a function of RWP and MTG treatments were monitored using sodium dodecylsulfate polyacrylamide gel electrophoresis (SDS-PAGE) as described by Laemmli (*18*), with some modifications proposed by Bulat and Topcu (*19*). Freeze-dried samples were dissolved in sample buffer (10 mg/mL) and 10 μL of sample were run through the stacking and separating gels at 200 mV. The gels were kept in fixing solution (*V*(TCA):*V*(methanol):*V*(deionized water)=1.0:3.3:5.7, *γ*(TCA)=g/mL) for 60 min. Then, the gels were stained overnight with Coomassie Brilliant Blue G-250 and destained in distilled water for 12 h. Electrophoretic studies were carried out by using Protean II XL vertical electrophoresis units (Bio-Rad Laboratories Ltd., Watford, UK).

### Determination of peptide profile

The samples were centrifuged (Sigma 3-18K; Sartorius AG, Göttingen, Germany) at 7500×*g* and 4 °C for 15 min to separate the liquid phase. A volume of 1 mL of the separated serum was taken and mixed with 1 mL of 0.2% trifluoroacetic acid (TFA) and filtered through a 0.45-µm membrane filter. The filtrate was injected into the HPLC (Thermo Finnigan Inc., San Jose, CA, USA) system coupled with an autosampler (model AS3000), a degaser (SCM 1000), a gradient pump (P4000) and a UV DAD detector (6000LP). The C18 (250 mm×4.6 mm, 5 µm, 300 Å) RP-HPLC column was used. Deionized water containing 0.1% TFA was used as mobile phase A and acetonitrile (UV grade) containing 0.1% TFA was used as mobile phase B (*19*). Measurements were made at 280 nm and data were evaluated with ChromQuest 5.0 software (Thermo Fisher Scientific Inc, Waltham, MA, USA).

### Sensory analysis

Sensory analyses of the ayran samples were performed using 7-member panel at the department of Dairy Technology at Ankara University. The 7 panellists (4 females and 3 males aged between 30 and 65) were selected from individuals who had previously received training on the sensory evaluation of fermented dairy products including ayran. The scoring card with a five-point hedonic scale (1=poor/unacceptable to 5=excellent/very much like) described by Clark and Costello (*20*) was used in the evaluations. Panellists were requested to evaluate the appearance, body, texture, aroma and flavour of the samples. Ayran samples were served to panellists at (4.0±0.5) °C. Three-digit random codes were used for each sample. In each session, four samples were presented to each panellist. Panellists were provided with bread sticks and a glass of water between each sample.

### Statistical evaluations

A randomized complete block design was used to analyse the response variables related to the characteristics of ayran samples. First, normality and homogeneity of variance tests were determined to see if ANOVA could be applied to the data. It was found that p-values in the normality test and significance values in the test of homogeneity of variance were higher than 0.05. After it was determined that the necessary conditions for the application of the ANOVA test were met, statistical evaluation of the study (ANOVA) was performed with Minitab^®^ package program, v. 16.1.1 (*21*). Tukey’s multiple-comparison test was used to determine the significantly different groups (p<0.05). The trials were run in duplicate.

## RESULTS AND DISCUSSION

### Variations in the gross composition of ayran samples

The gross chemical composition of ayran samples is presented in Table 1. The dry matter mass fractions of ayran samples were different from each other (p<0.05) because of the differences in the volumes of reconstituted whey powder solution (RWP) added to meet target protein content (2%, *m*/*V*) in the final products. The protein mass per volume ratio of the samples was between 1.98 and 2.04% (*m*/*V*). Since the protein content of the samples was standardized to 2%, we did not find any difference among them regarding total protein content (p>0.05). Similarly, the fat mass fractions of the samples were not different as they were adjusted to 1%. Interestingly, we expected to see differences among the samples regarding ash mass fraction, since we did not use demineralized whey powder and the volumes of the used RWP solution were different. Although a slight increase was noted with the increase in the volume of the added RWP solution, this was statistically insignificant (p>0.05). The pH values of the samples remained between 4.27 and 4.42 on day 1 and, as expected, decreased gradually as the storage time extended (Table S3). The addition of RWP solution slightly affected the pH values ​​of the samples (p>0.05). In line with the pH values, the total acidity values expressed as lactic acid (in %) varied within a narrow margin. The lactic acid values showed that the difference between control samples and samples A_10_, A_10+MTG_, A_15_ and A_15+MTG_ was significant. Depending on the amount of whey, the total acidity values differed. In general, as the whey amount increased, the total acidity value also increased. Since the addition of whey did not affect the number of microorganisms in ayran samples, the difference here was probably due to the acidity of the RWP solution. The addition of MTG had no effect either on the pH or total acidity values of the samples (p>0.05). In contrast to our findings, MTG was demonstrated to slow down the development of acidity in yogurt (*22*, *23*).

**Table 1:** Gross composition of ayran samples

Component	Sample							
Ctr 1	Ctr 2	A_5_	A_5+MTG_	A_10_	A_10+MTG_	A_15_	A_15+MTG_
*w*/%							
Dry matter	(6.92±0.06)^c^	(6.86±0.06)^c^	(8.0±0.5)^bc^	(7.9±0.4)^bc^	(9.5±0.3)^ab^	(9.4±0.2)^ab^	(10.6±0.6)^a^	(10.6±0.5)^a^
Protein	2.02±0.04	1.99±0.02	1.99±0.05	2.03±0.05	2.0±0.1	2.0±0.1	2.0±0.1	1.99±0.05
Fat	1.10±0.00	1.05±0.07	1.05±0.07	1.05±0.07	1.05±0.07	1.08±0.04	1.05±0.07	1.00±0.00
Salt	0.52±0.02	0.53±0.01	0.53±0.01	0.52±0.02	0.50±0.00	0.53±0.00	0.51±0.08	0.53±0.01
Ash	1.02±0.04	1.0±0.3	1.1±0.1	1.10±0.00	1.13±0.03	1.1±0.2	1.17±0.09	1.2±0.2

Ctr 1 and 2=samples without RWP and MTG (1) and with MTG (2) as control; A_5_, A_10_ and A_15_=samples with 5, 10 and 15% RWP; A_5+MTG_, A_10+MTG_ and A_15+MTG_=samples with 5, 10 and 15% RWP and 0.5 U/g MTG, RWP=reconstituted whey powder, MTG=microbial transglutaminase

### Microbiological profiles of ayran samples as affected by MTG and whey treatments

The counts of *S. thermophilus* and *L. bulgaricus* in ayran samples are given in Table 2. We noticed that the addition of RWP and MTG to milk for ayran production had no remarkable effect on either the fermentation profiles of the samples (data not shown) or the viability of *S. thermophilus* and *L. bulgaricus* (p>0.05). There is no consensus in the literature on the effects of MTG on the symbiotic growth of yogurt starter bacteria. Some researchers demonstrated that MTG-triggered cross-linking slightly imbalanced the protosymbiosis between yogurt starter cultures during fermentation (*24*, *25*), while others failed to observe any interference of MTG with the growth of yogurt starters and fermentation process (*26*, *27*). It is known that MTG does not have a direct effect on the growth and viability of yogurt starter bacteria. However, the new matrix formed by MTG through additional cross-linking of proteins may decelerate the development of acidity and hence fermentation. In the present case, it is thought that since ayran does not have a solid matrix, yogurt starters had no restrictions to reach lactose and ferment the milk without any major obstruction. Both bacterial counts did not show a significant change during storage (p>0.05). In addition, while the counts of *S. thermophilus* remained almost stable during storage period of 15 days, the number of *L. bulgaricus* colonies fluctuated slightly in the samples A_5+MTG_, A_15_ and control with MTG. Neve *et al.* (*25*) showed that the counts of bacteria in yogurt treated with MTG decreased (~0.5 log) during storage period of two weeks. On the other hand, Demirkaya and Ceylan (*23*) revealed that the counts of *L*. *bulgaricus* in yogurt treated with MTG increased, but the number of *S. thermophilus* decreased during 21 days of storage. On the contrary, Ziarno and Zareba (*28*) failed to demonstrate any impact of MTG on *L. bulgaricus,* but the growth of *S. thermophilus* showed a time-dependent variation during cold storage of yogurt. In the present case, the effect of MTG on the viability of yogurt bacteria was negligible. Özer *et al.* (*22*) demonstrated a negative relationship between the used MTG amount and counts of yogurt starter bacteria in stirred-type yogurt, being more pronounced at higher MTG contents. The same authors postulated that small molecular mass peptides required for the growth of *S. thermophilus* remained in the MTG-mediated gel matrix, which made them partly unavailable for yogurt bacteria (*22*). Ayran is a liquid product and protein cross-linking by MTG increases its viscosity rather than forming a 3-dimensional gel matrix as happens in yogurt. Therefore, it is thought that the presence of MTG did not hinder yogurt bacteria to reach small molecular mass peptides.

**Table 2:** Colony counts of *S. thermophilus* and *L. bulgaricus*

			Sample															
	*t*/day		Ctr 1		Ctr-2		A_5_		A_5+MTG_		A_10_		A_10+MTG_		A_15_		A_15+MTG_	
*N*/(log CFU/mL)																	
*S. thermophilus*																	
1		8.87±0.02		8.85±0.06		8.78±0.06		9.0±0.2		8.73±0.02		8.83±0.06		9.04±0.04		8.9±0.1	
7		8.92±0.06		8.82±0.02		8.8±0.1		8.60±0.06		8.94±0.03		9.03±0.04		8.9±0.1		8.8±0.1	
15		9.08±0.03		8.92±0.01		9.0±0.2		8.5±0.2		9.00±0.02		8.78±0.04		8.53±0.01		8.63±0.01	
*L. bulgaricus*																	
1		5.4±0.2		5.3±0.3		5.8±0.2		5.5±0.4		5.7±0.2		5.54±0.07		5.2±0.2		4.9±0.8	
7		5.3±0.1		6.7±0.4		5.7±0.5		4.8±0.3		5.0±0.4		5.51±0.06		6.6±0.8		5.3±0.2	
15		5.10±0.4		5.2±0.3		4.9±0.1		5.3±0.3		4.8±0.5		5.3±0.4		5.2±0.3		5.0±0.2	

Values are presented as mean±S.E (*N*=2). Non-lettering columns or rows indicate the differences between scores were not significant (p>0.05). Ctr 1 and 2=samples without RWP and MTG (1) and with MTG (2) as control; A_5_, A_10_ and A_15_=samples with 5, 10 and 15% RWP; A_5+MTG_, A_10+MTG_ and A_15+MTG_=samples with 5, 10 and 15% RWP and MTG, RWP=reconstituted whey powder, MTG=microbial transglutaminase

### Variation in colour properties of ayran samples

We found differences in the colour properties of the samples (p<0.01) (Table 3). The  -a* values (representing red-green colour) ​​of ayran samples increased with the increase in the amount of the added RWP (p<0.01). We observed that the both control samples (with and without MTG) were clearly different from the rest of the samples regarding -a* values. Whey has a greenish-yellow colour due to its high riboflavin content (*29*). For this reason, the samples supplemented with RWP were more greenish than the control samples. The -a* values ​​of ayran samples changed within a narrow margin during storage (p>0.05). The samples with added RWP at the highest amount (samples A_15_ and A_15+MTG_) differed from the other samples with regard to b* values (representing yellow-blue colour) ​​(p<0.01). Since the fat of the samples was standardized to 1% (*m/V*), the addition of RWP was the major factor causing colour differences among the samples. The lightness (L*) values of the samples decreased with the increase in the amount of RWP and this trend remained unchanged during storage. This may have stemmed from the decrease in casein amount of the samples containing high content of RWP. Caseins are primarily responsible for the opacity of milk. Samples A_15_ and A_15+MTG_ had lower Δ*E* and higher colour saturation (*C*) values. These results may well be due to the yellowness caused by higher lactose contents in these samples. Finally, all the colour parameters (L*, a* and b*) of ayran samples showed a significant difference (p<0.05) as the amount of whey was increased.

**Table 3:** Colour, Δ*E* and chroma (*C*) values of ayran samples

	*t*/day	Sample							
		Ctr 1	Ctr 2	A_5_	A_5+MTG_	A_10_	A_10+MTG_	A_15_	A_15+MTG_

	1	(96.0±0.6)^Ab*^	(96.2±0.5)^A^	(94.5±1.2)^B^	(93.1±3.5)^B^	(88.6±2.2)^C^	(88.9±3.7)^C^	(84.3±0.7)^D^	(83.2±2.6)^D^
L*	7	(96.1±0.6)^b^	96.1±0.4	93.1±0.3	92.5±0.7	90.0±0.7	89.0±1.9	85.0±0.6	86.8±0.8
	15	(94.9±0.5)^a^	96.1±0.6	93.3±0.3	93.5±2.0	92.2±0.3	92.5±0.9	88.9±0.5	90.2±0.4

	1	(8.4±0.2)^D^	(8.6±0.4)^D^	(11.9±1.1)^C^	(11.5±0.3)^C^	(16.5±0.3)^B^	(16.5±0.2)^B^	(18.9±1.1)^A^	(18.4±0.1)^A^
-a*	7	8.5±0.5	8.7±0.8	11.1±1.2	11.0±0.9	14.3±0.3	16.4±0.6	18.4±1.1	19.0±2.6
	15	8.5±0.2	8.6±0.4	11.3±0.0	11.3±0.3	14.5±0.3	15.1±0.9	18.0±0.7	17.7±0.9

	1	(7.6±0.3)^Cb^	(7.7±0.1)^C^	(7.6±0.4)^C^	(7.5±0.4)^C^	(9.6±0.3)^B^	(9.8±0.5)^B^	(13.3±0.1)^A^	(14.9±0.8)^A^
b*	7	(7.9±0.1)^b^	7.4±0.0	7.9±0.7	7.3±0.2	8.8±0.1	9.1±1.2	12.6±0.8	14.1±0.3
	15	(8.4±0.3)^a^	8.7±0.2	8.6±0.1	8.9±0.0	10.0±1.0	10.1±0.4	14.5±0.6	14.9±0.0

	1	(96.7±0.4)^A^	(96.9±0.4)^A^	(95.5±0.7)^A^	(94.1±2.4)^A^	(90.6±1.6)^B^	(91.0±2.6)^B^	(87.4±0.7)^C^	(86.5±1.7)^C^
Δ*E*	7	96.8±0.5	96.8±0.2	94.1±0.3	93.5±0.6	91.6±0.6	90.9±1.3	87.9±0.5	89.3±0.9
	15	95.7±0.4	96.9±0.5	96.4±0.2	94.6±1.4	93.8±0.1	94.3±0.5	91.8±0.3	93.2±0.1

	1	(11.3±0.3)^D^	(11.5±0.3)^D^	(14.2±0.8)^C^	(13.7±0.4)^C^	(19.1±0.3)^B^	(19.2±0.1)^B^	(23.0±0.7)^A^	(23.7±0.3)^A^
*C*	7	11.6±0.2	11.4±0.4	13.6±1.0	13.2±0.6	16.8±0.2	18.2±0.1	23.3±0.3	23.4±1.4
	15	11.9±1.1	12.2±0.3	14.2±0.1	13.6±0.2	18.3±0.6	18.1±0.7	22.7±0.2	23.2±0.5

Mean value±S.E. (*N*=2) in the same column with different lowercase letters show significant differences during storage (p<0.05). Mean values in the same row with different capital letters show significant differences among samples (p<0.05). Ctr 1 and 2=samples without RWP and MTG (1) and with MTG (2) as control; A_5_, A_10_ and A_15_=samples with 5, 10 and 15% RWP; A_5+MTG_, A_10+MTG_ and A_15+MTG_=samples with 5, 10 and 15% RWP and 0.5 U/g MTG; RWP=reconstituted whey powder; MTG=microbial transglutaminase

### Physical properties of ayran samples as affected by MTG and RWP combinations

We found that partial replacement of RWP in the production of ayran reduced the phase separation in the samples on day 1, being more pronounced with increased addition of water (Table 4). However, the phase separation values of the samples increased significantly throughout the storage period (p<0.05). We demonstrated that the addition of MTG prevented phase separation, which was slightly more pronounced in the samples containing RWP at higher amounts. Ayran has an unstable physical structure due to low pH values (pH=4.4-4.6) (*30*). The increase in phase separation during storage is explained by the increase in electrostatic force between casein micelles due to the addition of salt and decreasing pH value (*31*). Although the addition of RWP led to an increase in the dry matter of milk, the physical stability of the product became poorer. This may have resulted from the differences in the composition of the ayran samples, especially the differences in the balance of protein fractions and ionic salt amounts of milk and whey. The addition of MTG caused an increase in the consistency index (*K*) of ayran samples containing RWP (Table 4). On the other hand, the addition of reconstituted whey powder alone or in combination with MTG resulted in decreases of *K* values compared to the both control samples. These results were further confirmed by phase separation results. During storage, the consistency index of the experimental samples increased.

**Table 4:** Physical properties of ayran samples

	Sample							
	Ctr 1	Ctr 2	A_5_	A_5+MTG_	A_10_	A_10+MTG_	A_15_	A_15+MTG_
Phase separation/% *t*/day								
1	(5.5±0.7)^g^	(6.0±0.0)^g^	(5.5±0.7)^g^	(6.0±1.4)^g^	(3.5±0.7)^g^	(3.5±0.7)^g^	(3.0±0.0)^g^	(2.0±0.0)^g^
7	(20.0±0.0)^de^	(17.5±0.7)^ef^	(28.0±0.0)^abc^	(29.5±0.7)^ab^	(24.0±1.4)^cd^	(13.0±1.4)^f^	(28.0±1.4)^abc^	(18.5±0.7)^e^
15	(21.5±2.1)^de^	(20.5±2.1)^de^	(31.0±1.4)^ab^	(30.5±0.7)^ab^	(28.5±0.7)^abc^	(21.5±2.1)^de^	(32.5±2.1)^a^	(27.0±0.0)^bc^
*K*/(Pa⋅s)								
1	(0.5±0.2)^bc^	(0.7± 0.2)^a^	(0.37±0.07)^bcd^	(0.57±0.05)^ab^	(0.3±0.2)^d^	(0.39±0.05)^bcd^	(0.22±0.07)^d^	(0.30± 0.05)^cd^
7	0.59±0.01	1.0±0.1	0.46±0.03	0.7±0.1	0.30±0.06	0.40±0.07	0.22±0.08	0.28±0.01
15	0.63±0.06	0.83±0.02	0.40±0.06	0.6±0.1	0.3±0.1	0.53±0.07	0.3±0.2	0.34±0.00
*n*								
1	(0.32±0.02)^a^	(0.34±0.04)^a^	(0.38±0.02)^ab^	(0.34±0.02)^ab^	(0.41±0.06)^ab^	(0.32±0.06)^ab^	(0.44±0.06)^b^	(0.38±0.05)^ab^
7	0.31±0.03	0.28±0.02	0.34±0.02	0.31±0.03	0.36±0.05	0.36±0.04	0.40±0.05	0.38±0.02
15	0.28±0.01	0.31±0.00	0.33±0.01	0.32±0.02	0.38±0.05	0.34±0.04	0.39±0.09	0.37±0.01

Mean value±S.E (*N*=2) with different letters show significant differences among values (p<0.05). Ctr 1 and 2=samples without RWP and MTG (1) and with MTG (2) as control; A_5_, A_10_ and A_15_=samples with 5, 10 and 15% RWP; A_5+MTG_, A_10+MTG_ and A_15+MTG_=samples with 5, 10 and 15% RWP and 0.5 U/g MTG; RWP=reconstituted whey powder; MTG=microbial transglutaminase

Similar results were obtained for the flow behaviour index (*n*) of the samples. The addition of RWP and MTG did not cause any remarkable change in the flow type of ayran. Flow behaviour index of the samples was ​​between 0.32 and 0.44 on day 1 and between 0.28 and 0.39 on day 15, and ayran samples showed a pseudoplastic flow behaviour throughout storage period (Table 4). The only negative effect of using RWP in ayran production was on the physical properties of the samples. This handicap was overcome to some extent with MTG treatment. The increase in viscosity and decrease in time-dependent sedimentation in ayran treated with MTG was reported by Şanli *et al*. (7) and Rezazadeh-Bari *et al*. (*8*). The texture-enhancing properties of MTG in yogurt (*28*, *32*, *33*) and in kombucha fermented milk (*34*) have also been reported.

### Variations in volatile profiles of ayran samples

Volatile compounds of the samples are given in Table 5. Acetaldehyde, acetoin and diacetyl were the most abundant carbonyl compounds in all samples. We found that the acetaldehyde concentrations in the samples varied within narrow margins on each storage day and neither the addition of RWP nor MTG affected them significantly (p>0.05). On the other hand, acetaldehyde concentrations in the samples decreased significantly during storage (p<0.05). The time-dependent decrease in acetaldehyde concentrations in ayran may be attributed to the oxidation of acetaldehyde to acetate (*15*) or degradation of acetaldehyde to alcohol by alcohol dehydrogenase (*2*). It is known that very few strains of yogurt bacteria have an alcohol dehydrogenase, and we have no information whether the starter bacteria used in the present study had such enzyme activity. On the other hand, since ethanol was not detected in any of the samples, it may be thought that the commercial yogurt starter strains were alcohol dehydrogenase-negative. Acetaldehyde is the most characteristic carbonyl compound of yogurt and is found in yogurt at levels varying from 2 to 40 mg/L (*15*). For a yogurt with a well-balanced aroma and flavour, the acetaldehyde concentration should be between 15 and 30 mg/L, and characteristic yogurt aroma could hardly be perceived at acetaldehyde concentration of ≤4 mg/L (*2*).

**Table 5:** Major volatile compounds of ayran samples

	Sample							
	Ctr	Ctr 2	A_5_	A_5+MTG_	A_10_	A_10+MTG_	A_15_	A_15+MTG_
*γ*(carbonyl compound)/(mg/L) *t*/day								
Acetaldehyde								
1	(14.4±1.2)^a^	16.0±4.2	15.6±2.7	12.0±2.5	15.6±1.5	14.8±1.6	12.9±1.6	12.3±0.8
7	(13.0±1.2)^ab^	14.1±0.4	15.0±1.3	10.9±0.9	14.0±0.2	14.3±0.7	12.3±0.9	10.3±0.3
15	(10.1±0.6)^b^	12.7±1.2	11.1±1.1	13.1±0.8	12.3±0.9	15.5±4.2	12.8±1.7	10.9±1.4
Diacetyl								
1	9.6±1.3	15.9±1.5	13.1±0.4	10.4±1.0	15.34±1.8	13.7±1.1	16.8±0.5	11.9±0.6
7	11.8±1.5	19.5±0.7	17.5±3.0	17.1±1.8	15.9±3.5	14.1±4.9	11.6±1.4	11.4±1.2
15	11.4±1.5	9.9±1.8	14.3±1.4	12.8±2.4	10.1±0.4	14.4±1.6	11.7±1.8	13.1±1.1
Acetoin								
1	(14.8±1.0)^a^	19.8±1.5	22.4±2.1	21.7±1.6	25.2±1.4	23.1±3.5	17.9±2.8	17.4±2.2
7	(21.7±1.8)^b^	20.0±2.4	19.7±1.6	21.1±3.3	28.4±1.9	26.2±3.3	26.3±3.6	24.0±3.4
15	(14.9±0.4)^c^	13.0±0.9	15.0±0.6	11.6±1.8	14.7±1.5	13.6±1.2	10.5±0.6	10.3±0.7
*γ*(compound other than carbonyl)/(mg/L)								
Hexenal								
1	(17.7±1.6)^a^	20.9±0.8	20.4±2.0	22.0±1.6	25.2±1.4	23.6±1.7	14.0±3.1	19.6±4.7
7	(14.9±1.1)^b^	14.4±1.5	10.5±0.5	18.3±3.2	12.9±2.5	15.7±1.9	12.4±1.4	14.1±2.3
15	(13.9±0.3)^c^	10.9±0.8	12.2±0.4	13.1±0.5	12.6±0.6	8.0±0.6	9.3±1.5	12.6±0.5
2,3-Pentanedione								
1	(10.1±3.5)^b^	11.8±1.9	10.8±0.3	11.8±1.34	15.6±4.1	14.0±1.6	13.2±2.6	12.8±2.0
7	(15.0±3.5)^a^	15.3±0.7	13.6±1.5	16.0±3.3	16.3±5.7	15.0±2.7	18.0±3.4	16.1±5.8
15	(9.1±0.9)^b^	6.2±1.0	10.0±0.5	11.6±1.9	13.1±0.3	9.4±0.6	10.80±0.04	11.8±1.6
Benzaldehyde								
1	nd^b^	nd	nd	nd	4.6±1.4	1.9±0.5	nd	nd
7	nd^b^	nd	nd	1.5±0.2	nd	3.1±0.5	2.9±0.4	nd
15	(2.1±0.2)^a^	4.1±0.5	2.0±0.2	2.1±0.3	4.6±1.3	2.8±1.9	4.3±1.6	nd
2-Octanone								
1	(1.1±0.3)^b^	2.0±1.2	2.2±0.7	1.6±0.2	1.33±0.48	3.4±2.1	2.3±0.8	2.8±1.6
7	(6.4±0.2)^a^	5.3±0.9	6.0±1.1	5.92±0.04	6.5±0.2	9.4±1.2	6.6±2.0	6.2±0.9
15	(1.70±0.03)^b^	1.63±0.07	4.5±0.9	3.5±2.5	1.0±0.2	1.6±0.7	4.4±1.2	2.1±0.2
2-Nonanone								
1	nd^b^	1.15±0.06	nd	1.3±0.3	0.2±0.2	1.5±0.1	0.99±0.02	1.1±0.1
7	nd^b^	nd	nd	1.5±0.3	2.6±0.8	3.3±1.0	1.4±0.4	nd
D15	(1.3±0.3)^a^	1.2±0.2	3.0±0.2	nd	2.9±0.5	2.2±1.0	2.7±0.3	nd
2-Undecanone								
1	nd^b^	nd	nd	nd	nd	nd	nd	nd
7	nd^b^	nd	nd	nd	nd	nd	nd	nd
15	(1.10±0.09)^a^	1.5±0.5	2.5±0.8	1.5±0.3	1.3±0.5	2.0±1.4	1.56±1.9	1.5±0.1
Acetic acid								
1	(1.14±0.07)^ab^	0.70±0.03	1.4±0.9	0.6±0.4	0.3±0.1	0.4±0.1	1.21±0.09	1.3±0.2
7	(1.21±0.07)^a^	0.9±0.2	0.7±0.2	0.8±0.5	2.6±2.1	2.6±1.0	1.22±0.62	0.7±0.2
15	(1.4±0.1)^b^	0.75±0.04	0.7±0.5	0.9±0.1	0.27±0.05	0.5±0.3	0.34±0.07	1.0±0.7
Ethanol								
1	nd^b^	nd	nd	nd	nd	nd	nd	nd
7	nd^b^	nd	nd	nd	nd	nd	nd	nd
15	(0.4±0.2)^a^	nd	0.23±0.01	0.98±0.08	0.79±0.09	nd	nd	nd

Mean value±S.E. (*N*=2) in the same column with different letters show significant differences during storage (p<0.05). Ctr 1 and 2=samples without RWP and MTG (1) and with MTG (2) as control; A_5_, A_10_ and A_15_=samples with 5, 10 and 15% RWP; A_5+MTG_, A_10+MTG_ and A_15+MTG_=samples with 5, 10 and 15% RWP and 0.5 U/g MTG; RWP=reconstituted whey powder; MTG=microbial transglutaminase; nd=not detected

Diacetyl (2,3-butanedione), which is responsible for a buttery taste in dairy products, has a limited effect on the aroma of yogurt. However, when acetaldehyde is present at low concentrations in yogurt, the influence of diacetyl on aroma increases. Diacetyl concentrations in the samples did not differ during storage and were found to be independent from RPW and MTG treatments (p>0.05). Similar findings were reported by Şanli *et al*. (*7*) as well. Except for the samples A_15_ and A_15+MTG_, diacetyl concentrations in the rest of the samples increased slightly within the first week of storage and then decreased again at the end of storage.

Acetoin (2-butanone-3-hydroxy) contributes to the characteristic flavour of yogurt together with acetaldehyde, acetone and diacetyl. Variations in the acetoin concentrations in the samples during storage were more or less similar to that of diacetyl. With the exception of samples A_5_ and A_5+MTG_, we observed increases in the acetoin concentrations in the samples within the first seven days of storage (p<0.01) and then a sharp decline was noted in all samples on day 15 (p<0.01). Slightly lower acetoin concentrations were obtained in the samples with added RWP at the highest amount (samples A_15_ and A_15+MTG_) on day 15. The effects of both RWP and MTG on the acetoin concentrations were found to be negligible (p>0.05). Both diacetyl and acetoin are formed by fermenting citrate by lactic acid bacteria during pyruvate metabolism (*2*). Acetoin is usually present in yogurt at higher concentrations than diacetyl (*35*).

Hexanal and 2,3-pentanedione were the most abundant volatile compounds other than carbonyls in all samples (Table 5). Hexanal is a straight-chain aldehyde type aroma component characterized by fruity flavour and is produced by β-oxidation of unsaturated fatty acids (*36*). The concentration of hexanal decreased in ayran samples during storage (p<0.01), possibly due to its rapid degradation. Similar results were reported by Gassenmeier (*37*) who showed a rapid decrease of hexanal in yogurt within the first 5 days of storage. The 2,3-pentanedione is formed by aldol condensation of α-oxobutyric acid formed by the breakdown of threonine amino acid (*38*) and is primarily responsible for buttery, caramelized and vanilla aromas (*39*). No remarkable differences were noted in 2,3-pentanedione concentration in the samples on each storage day (p>0.05). However, we noted fluctuations in the concentrations of this volatile compound in all samples throughout storage (p<0.01). The 2-octanone and acetic acid concentrations of the samples also fluctuated during storage (p<0.01). The 2-nonanone was found at fairly low concentrations in ayran samples. Apart from these volatile compounds, benzaldehyde, ethanol and 2-undecanone were detected at very low concentrations on day 15 but were not detected in the samples on days 1 and 7.

### Electrophoretic patterns of proteins in ayran samples

**Fig. 1:**
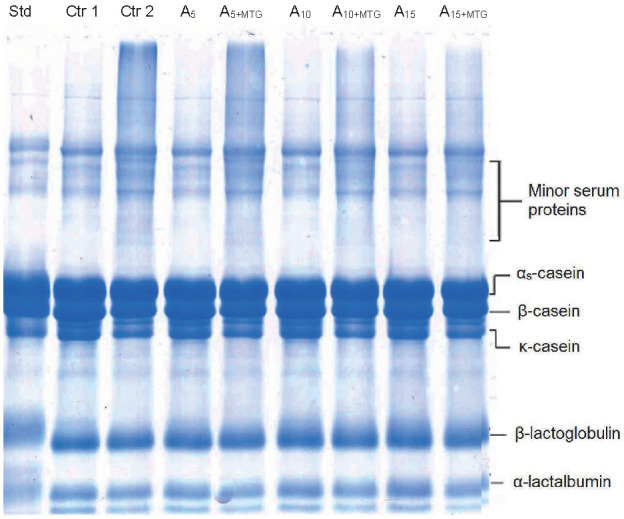
Sodium dodecyl sulfate–polyacrylamide gel electrophoresis (SDS-PAGE) electrophoretogram of 15-day-old ayran samples. Std=standard; Ctr 1 and 2=samples without RWP and MTG (1) and with MTG (2) as control; A_5_, A_10_ and A_15_=samples with 5, 10 and 15% RWP; A_5+MTG_, A_10+MTG_ and A_15+MTG_=samples with 5, 10 and 15% RWP and MTG, RWP=reconstituted whey powder, MTG=microbial transglutaminase

SDS-PAGE electrophoretograms of the samples are given in Fig. 1. We observed no clear differences in electrophoretic patterns of the samples without MTG (samples Ctr 1, A_5_, A_10_ and A_15_). On the other hand, in the sample group supplemented with whey and treated with MTG (samples A_5+MTG_, A_10+MTG_ and A_15+MTG_), the bands representing cross-linked aggregates became more visible. This coincided with decrease in the intensities of the bands representing β-lactoglobulin (β-Lg) and α-lactalbumin (α-La) in samples A_5+MTG_, A_10+MTG_ and A_15+MTG_. In a study investigating the digestibility of cross-linked proteins, high molecular mass polymers were detected in the upper parts of the band (especially above 30 kDa) in the samples treated with MTG (*40*). In the present case, such dense bands were also evident in MTG-treated samples (Fig. 1). However, cross-linked aggregate formation decreased with increasing the amount of whey protein. Especially in A_15+MTG_ sample, this was more visible. We noted no clear degradation in protein fractions during storage (not shown). Although the proteolytic capacity of yogurt starter bacteria is strain-dependent, majority of the commercial yogurt starter strains are known to be weakly or moderately proteolytic. Therefore, a high degree of proteolysis in yogurt-type fermented products such as ayran is not expected.

### Variations in the peptide profiles of ayran samples

**Fig. 2:**
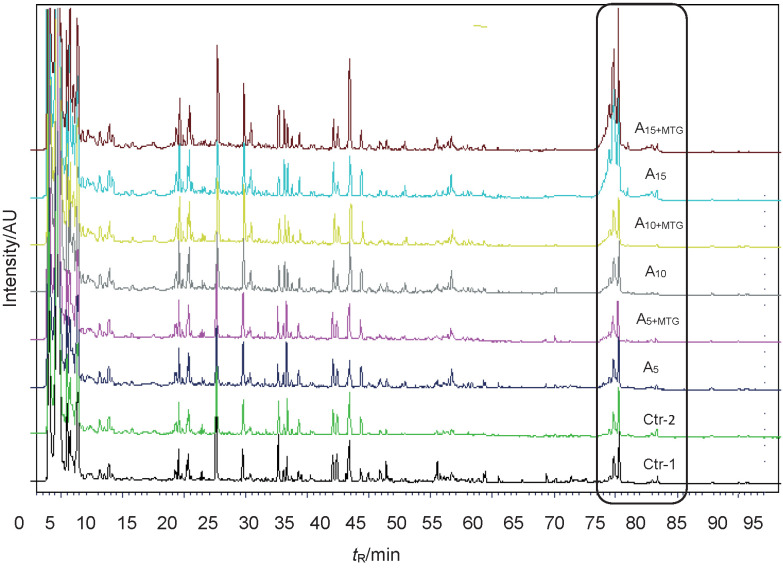
Variation in the peptide profile of 15-day-old ayran samples determined by HPLC. A_5_, A_10_ and A_15_=samples with 5, 10 and 15% RWP; A_5+MTG_, A_10+MTG_ and A_15+MTG_=samples with 5, 10 and 15% RWP and MTG; Ctr 1 and 2=samples without RWP and MTG (1) and with MTG (2) as control; RWP=reconstituted whey powder; MTG=microbial transglutaminase

Peptide profiles of the experimental samples are presented in Fig. 2. We did not observe differences in the hydrophilic peptides within the first 30 min of the chromatograms (tyrosine-tryptophan region) between the ayran samples with added whey and MTG. As expected, the amount of serum proteins such as α-lactalbumin and β-lactoglobulin (late eluting peaks after 80 min, Fig. 2) was found to be higher in the ayran produced with whey, depending on the increased amount of whey. In addition, in the samples with less whey, similar amounts of early eluting peptides and late eluting peaks were observed. However, as the amount of whey increased, the late eluting peaks were significantly higher than the early peaks. The addition of the MTG had no significant effect on peptide profiles of the samples. However, the peaks representing β-Lg were found to be smaller in the samples treated with MTG. Regarding hydrophobic peptides, which were represented by the peaks that appeared on the chromatograms after 55 min, no clear difference was detected between the samples. In addition, no difference was found in the peptide profiles of the samples during storage (not shown). Yuan *et al*. (*41*) found that the antioxidant acitivity in MTG-treated yogurt was higher than in the untreated yogurt and MTG altered the peptide profiles of yogurt, evidenced by the release of higher amount of low molecular mass (<1.5 kDa) peptides and amino acids. The same authors demonstrated that peptides in the MTG-treated yogurts were released mainly from κ-caseins as opposed to the control yogurt in which β-casein was the main source of small molecular mass peptides.

### Impact of utilization of MTG and reconstituted whey on sensory scores of ayran samples

**Fig. 3:**
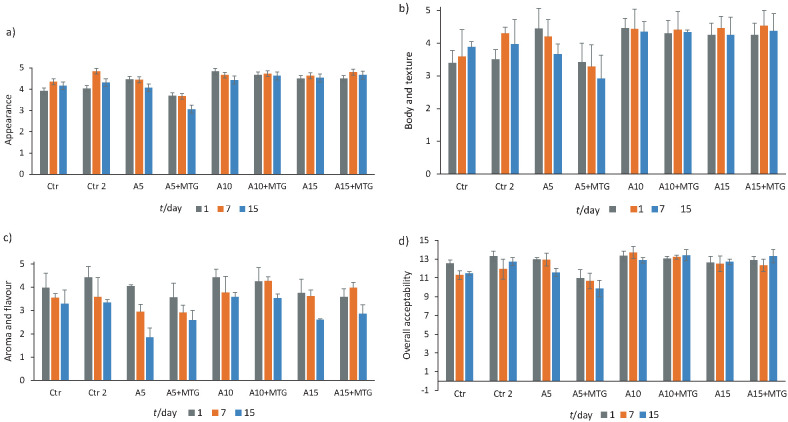
Sensory scores of ayran samples: a) appearance, b) body and texture, c) aroma and flavour, and d) overall acceptability. Different lowercase letters show significant differences among samples (p<0.05). In d) the differences between scores are not significant (p>0.05). Ctr 1 and 2=samples without RWP and MTG (1) and with MTG (2) as control; A_5_, A_10_ and A_15_=samples with 5, 10 and 15% RWP; A_5+MTG_, A_10+MTG_ and A_15+MTG_=samples with 5, 10 and 15% RWP and % MTG; RWP=reconstituted whey powder; MTG=microbial transglutaminase

One of the main goals of product development studies is to gain high consumer appreciation. In order to see if partial replacement of milk with whey in ayran production had any effect on the sensory properties of the resulting products, the samples were subjected to sensory analysis. Regarding appearance, body and texture properties of the samples, the incorporation of whey and MTG in ayran had positive effects (Figs. 3a and 3b). The appearance, body and texture scores of the control sample (Ctr 1) were lower than of the samples with added MTG, except for sample A_5+MTG_. Flavour scores indicated that the addition of whey and MTG did not have negative effect on the aroma and flavour of ayran (Fig. 3c). The storage period did not affect the sensory properties of the samples (p>0.05). All the samples supplemented with MTG and/or RWP, with the exception of A_5+MTG_, had higher overall sensory acceptance scores than the control (Ctr 1) on day 15. None of the panellists reported an unacceptable aroma, flavour, body, texture and appearance defects for any of the samples. The sensory results showed that the addition of whey has a positive effect on the sensory properties of ayran samples. All sensory results were better in the samples containing 10% or more whey (Fig. 3).

## CONCLUSIONS

In this study, total protein content of ayran samples (5, 10 or 15%) was accomplished by the addition of reconstituted whey powder (RWP) solution which was used to dilute the milk in ayran production. The addition of RWP did not affect the overall sensory acceptance of the resulting product, but physical stability of ayran slightly deteriorated and time-dependent sedimentation in the samples was evident during cold storage. This adverse effect was largely compensated by adding 0.5 U per g protein of microbial transglutaminase. Partial replacement of milk with RWP solution as diluting medium had no impact on the development of acidity and formation of volatile components in ayran. Results revealed that the addition of 10% RWP to the final product (samples A_10_ and A_10+MTG_) would be applicable in industrial production of ayran. Although the economical part of using RWP in ayran production was not evaluated here, it is envisaged that this strategy would lead to a reduction in the ayran production cost since it requires a smaller volume of milk. This model also offers a new way of valorisation of cheese whey. Future studies should focus on the determination of shelf life of ayran with added RWP, as well as the economic feasibility at medium and large-scale productions.

## Figures and Tables

**Table S1:** Phase separation and sensory results of preliminary trials

Sample	*t*/day	Phase separation/%	Appearance	Body and texture	Aroma and flavour
Ctr 1	1	11.5±2.0	5.0±0.0	4.6±0.34	4.1±0.2
	7	25.0±3.5	4.9±0.3	4.2±0.3	4.0±0.3
	15	27.5±4.5	4.6±0.2	4.2±0.3	4.0±0.5
MG-0	1	10.0±2.5	4.5±0.3	4.5±0.3	4.4±0.4
	7	22.0±4.0	4.8±0.4	4.5±0.5	4.0±0.4
	15	23.0±2.0	4.5±0.3	4.0±0.3	2.7±0.2
MTG-0.5	1	2.50±0.5	5.0±0.0	4.7±0.3	4.6±0.5
	7	10.0±3.0	5.0±0.0	4.6±0.3	4.3±0.3
	15	18.5±3.5	4.7±0.2	4.8±0.4	4.3±0.2
MTG-1.0	1	2.8±1.3	5.0±0.0	4.6±0.3	4.5±0.3
	7	13.0±2.0	5.0±0.0	4.5±0.3	4.1±0.3
	15	20.0±2.5	4.8±0.7	4.7±0.3	4.0±0.2
MTG-1.5	1	6.5±1.5	4.4±0.2	4.0±0.5	4.4±0.2
	7	16.0±2.0	4.5±0.4	4.3±0.4	4.1±0.4
	15	21.5±3.5	4.6±0.2	4.1±0.4	3.4±0.2

Values are presented as mean±S.E. (*N*=2). Ctr 1=control sample without RWP or MTG; MTG-0, MTG-0.5 and MTG-1.0 and MTG-1.5=ayran sample with 0.0, 0.5, 1.0 and 1.5 U per g MTG; MTG=microbial transglutaminase

**Table S2:** Sample composition and descriptions

**Sample**	(*m*(RWP)/*V*(solution))/%	Protein source		Activity(MTG)/(U/g)
(*m*(milk protein)/(*V*(solution))/% (*m*(RWP)/*V*(solution))/%	
**Ctr 1**	0	2	-	-
**Ctr 2**	0	2	-	0.5
**A_5_**	5	1.9	0.1 *	-
**A_10_**	10	1.8	0.2 **	-
**A_15_**	15	1.7	0.3 ***	-
**A_5+MTG_**	5	1.9	0.1 *	0.5
**A_10+MTG_**	10	1.8	0.2 **	0.5
**A_15+MTG_**	15	1.7	0.3	0.5

*V*(solution)=100 mL (milk with different additions of water). Ctr 1 and 2=samples without RWP and MTG (1) and with MTG (2) as control; A_5_, A_10_ and A_15_=samples with 5, 10 and 15% RWP; A_5+MTG_, A_10+MTG_ and A_15+MTG_=samples with 5, 10 and 15% RWP and 0.5 U/g MTG; RWP=reconstituted whey powder; MTG=microbial transglutaminase

**Table S3:** Acidity of ayran samples

*t*/day	Sample							
Ctr 1	Ctr 2	A_5_	A_5+MTG_	A_10_	A_10+MTG_	A_15_	A_15+MTG_
pH value									
1	4.28±0.05	4.27±0.04	4.34±0.09	4.40±0.01	4.40±0.04	4.42±0.06	4.39±0.10	4.42±0.10
7	4.13±0.07	4.11±0.05	4.18±0.09	4.22±0.05	4.21±0.08	4.23±0.11	4.27±0.13	4.26±0.11
15	4.12±0.08	4.08±0.04	4.15±0.06	4.17±0.00	4.16±0.01	4.16±0.00p	4.19±0.05	4.19±0.06
Titratable acidity (as lactic acid)/%									
1	(0.48±0.01)^a^	(0.47±0.00)^a^	(0.49±0.01)^a^	(0.49±0.03)^a^	(0.53±0.05)^b^	(0.53±0.05)^b^	(0.55±0.05)^b^	(0.55±0.06)^b^
7	0.51±0.01	0.51±0.02	0.54±0.06	0.52±0.04	0.59±0.04	0.60±0.04	0.61±0.06	0.61±0.06
15	0.52±0.05	0.52±0.04	0.56±0.07	0.54±0.07	0.60±0.05	0.61±0.06	0.61±0.09	0.63±0.07

Mean value±S.E. (*N*=2) in the same row with different letters show significant differences among samples (p<0.05). Ctr 1 and 2=samples without RWP and MTG (1) and with MTG (2) as control; A_5_, A_10_ and A_15_=samples with 5, 10 and 15% RWP; A_5+MTG_, A_10+MTG_ and A_15+MTG_=samples with 5, 10 and 15% RWP and 0.5 U/g MTG; RWP=reconstituted whey powder; MTG=microbial transglutaminase
